# Virtual screening of marine coumarins and xanthenes identifies novel acid-suppressive leads targeting histamine H_2_ receptor and gastric proton pump

**DOI:** 10.17305/bb.2026.13660

**Published:** 2026-01-28

**Authors:** Amar Osmanović, Mirsada Salihović, Amila Mehmedović, Amila Turalić, Elma Veljović, Mirha Pazalja, Simone Carradori, Selma Špirtović-Halilović

**Affiliations:** 1 University of Sarajevo, Faculty of Pharmacy, Sarajevo, Bosnia and Herzegovina; 2 Gastroenterohepatology Clinic, Clinical Center of University of Sarajevo, Sarajevo, Bosnia and Herzegovina; 3 University of Sarajevo, Faculty of Health Studies, Sarajevo, Bosnia and Herzegovina; 4 Department of Pharmacy, “G.d’Annunzio” University of Chieti-Pescara, Chieti, Italy

**Keywords:** Marine compounds, coumarin, xanthene, histamine H_2_ receptor, gastric H^+^/K^+^-ATPase, virtual screening

## Abstract

Marine natural products represent a diverse collection of structurally distinct metabolites, many of which have untapped therapeutic potential. This study screened 161 marine-derived coumarin and xanthene compounds for their binding affinity to the histamine H_2_ receptor and the gastric H^+^/K^+^-ATPase, the primary regulators of gastric acid secretion. Docking simulations were performed using curated structures of both targets, followed by an evaluation of the compounds for drug-likeness and predicted absorption, distribution, metabolism, and excretion (ADME) properties. Thirty-four compounds demonstrated a stronger predicted affinity for the H_2_ receptor than famotidine; however, only three compounds (1, 5, and 150) met all drug-likeness criteria, achieving quantitative estimates of drug-likeness (QED) values exceeding 0.67. Screening against the proton pump yielded 98 hits with higher affinity than soraprazan, with compound 150 being the only candidate to fulfill all medicinal chemistry filters. Interaction analysis indicated that compound 150 binds to the proton pump in a manner that largely overlaps with soraprazan. Density functional theory (DFT) calculations were utilized to characterize the electronic properties of the most promising compounds. ADME predictions suggested favorable permeability and a low risk for human ether-à-go-go-related gene (hERG) inhibition, although high plasma protein binding and the potential for cytochrome P450 (CYP) inhibition may require further optimization. These findings underscore the potential of pyranocoumarin compound 150, along with xanthene derivatives 1 and 5, as promising candidates for the development of new acid-suppressive agents.

## Introduction

Normal gastric acid secretion is essential for digestion and protects the stomach from pathogens. However, excessive or dysregulated acid production contributes to several common gastrointestinal disorders. The primary causes of hypersecretion include *Helicobacter pylori* infection, gastrin-secreting tumors associated with Zollinger–Ellison syndrome, and antral G-cell hyperplasia. These conditions operate through different mechanisms to overstimulate gastric parietal cells [1]. Persistent acid overproduction is central to the pathogenesis of gastroesophageal reflux disease (GERD) and peptic ulcer disease, potentially leading to Barrett’s esophagus and esophageal adenocarcinoma [2]. Dietary factors also significantly influence gastric acid handling. For instance, high-protein meals and certain beverages, such as milk and fermented drinks, tend to increase acid production. Conversely, heavy or high-fat foods may slow gastric emptying, prolonging gastrin levels and resulting in continued acid release. Additionally, strongly seasoned foods can irritate the mucosa, further stimulating acid production [3, 4].

The management of acid-related disorders typically involves strategies to either reduce acid production or neutralize existing gastric acid. Neutralization is achieved with antacids, which include inorganic salts like sodium bicarbonate, magnesium hydroxide, and calcium carbonate, as well as bismuth or aluminum-containing compounds such as silicates. These agents buffer or adsorb excess gastric acid. In contrast, acid-suppressive therapy employs drugs that inhibit acid secretion, most notably histamine H_2_ receptor antagonists (H_2_RAs) and proton pump inhibitors (PPIs) [2, 5].

H_2_RAs reduce gastric acid by blocking histamine binding to H_2_ receptors on parietal cells, thereby lowering both resting acid output and the postprandial surge. Famotidine remains commonly used, although its efficacy can diminish with prolonged use due to the development of tachyphylaxis [6, 7]. Ranitidine was once widely prescribed; however, it has largely been withdrawn from clinical use following the discovery of *N*-nitrosodimethylamine (NDMA) in multiple formulations, a compound classified as probably carcinogenic to humans [8].

PPIs, such as omeprazole, lansoprazole, and pantoprazole, provide more potent and longer-lasting acid suppression by irreversibly inhibiting the gastric H^+^/K^+^-ATPase. While generally effective, they are associated with certain drawbacks. Their onset of action is slower, individual responses may vary, and long-term use raises concerns about potential risks, including *C. difficile* infections, chronic kidney disease, and various nutrient deficiencies [9–12]. Additionally, practical issues arise, as the absorption of PPIs can be delayed when taken with food, reducing their bioavailability, while H_2_RAs often require careful timing around meals or known triggers [13].

A newer class of medications, potassium-competitive acid blockers (P-CABs) such as soraprazan, vonoprazan, and tegoprazan, presents another therapeutic option. These drugs inhibit the proton pump by competing at the potassium-binding site and generally provide faster and more consistent acid suppression compared to older therapies [9, 10, 14]. Despite their growing use, these medications have recognized limitations, and their long-term safety profiles are still being established, warranting continued exploration of novel therapeutic approaches.

Coumarins and xanthenes have been extensively studied as natural-product scaffolds, with their core structures remaining attractive starting points in drug discovery. The characteristic benzopyrone and dibenzo-*γ*-pyrone ring systems confer a relatively rigid, planar structure to these molecules, with carbonyl and phenolic oxygen atoms arranged in ways that significantly influence their polarity, hydrogen-bonding behavior, and overall lipophilicity. These features help explain the broad spectrum of biological activities reported for both classes and their association with drug-like compounds. Consequently, coumarins and xanthenes are often regarded as “privileged” scaffolds and are commonly employed as starting points in early lead discovery and optimization efforts [15–18].

Marine-derived metabolites further expand this diversity. Marine organisms, primarily fungi and bacteria, produce numerous coumarin and xanthene derivatives, many of which exhibit antimicrobial, anti-inflammatory, or anticancer properties [19]. Secondary metabolites, including coumarins and xanthenes, are typically isolated through fermentation of the producing organism followed by ethyl acetate extraction of the culture broth and biomass, with subsequent chromatographic purification to yield the target compounds [20–22]. The range of biological activities suggests that marine-derived compounds from these structural families may represent promising candidates for further investigation. Xanthenes, particularly their close structural relatives, xanthones, are often implicated in antisecretory or cytoprotective pathways [23–26]. While studies specifically examining marine-derived coumarins and xanthenes in gastric acid suppression are limited, research on plant-derived analogues provides useful context. Several coumarins of terrestrial origin have been reported to inhibit the gastric H^+^/K^+^-ATPase, with IC_50_ values ranging from approximately 110–638 µM. Xanthenes have demonstrated similar *in vitro* activity, with IC_50_ values ranging from 47 µM to 1.6 mM, indicating their potential as acid-suppressive compounds [27]. Additionally, various coumarin derivatives exhibit gastroprotective properties, including urease inhibition against *Helicobacter pylori* and measurable anti-ulcer effects [28]. These findings suggest that marine-derived coumarin and xanthene structures warrant further examination in the context of gastric acid–related conditions.

Computational methods have become increasingly central in early-stage drug discovery. Molecular docking is routinely employed to explore potential interactions between candidate ligands and their intended targets, estimating binding affinities. Concurrently, assessments of drug-likeness, utilizing criteria such as Lipinski’s, Veber’s, and Egan’s rules, along with metrics like the quantitative estimate of drug-likeness (QED), provide a practical means to evaluate the pharmacokinetic and overall development potential of new compounds [29–32]. Moreover, density functional theory (DFT) calculations are often utilized to investigate the electronic properties of drug-like candidates, complementing the interpretation of ligand–target interactions. Thus, *in silico* screening of marine-derived coumarin and xanthene derivatives against both the histamine H_2_ receptor and the gastric proton pump represents a rational strategy to identify candidates capable of overcoming the limitations of existing therapies.

## Materials and methods

### Virtual screening

A total of 161 compounds, focusing on xanthene and coumarin scaffolds isolated from marine organisms [20–22, 33–58], were selected for *in silico* screening. These derivatives were identified through a systematic literature search aimed at maximizing structural diversity within these classes. During the selection process, compounds with previously reported cytotoxicity or established toxicological liabilities (e.g., aflatoxins) were deliberately excluded to prioritize those with the greatest potential for therapeutic safety. Additionally, the reference ligands famotidine, a histamine H_2_ receptor antagonist, and soraprazan, a potassium-competitive acid blocker, were included in the library. Ligand 3D conformations were generated from Simplified Molecular Input Line Entry System (SMILES) strings and prepared using the AutoSMILES protocol in YASARA Structure v23.9.29 [59, 60]. To ensure accurate docking precursors, protonation states were assigned based on a physiological pH of 7.4 by predicting pK_a_ values and optimizing the hydrogen-bonding network through the experimental neutralization procedure. This protocol identified the most energetically favorable tautomeric states at the specified pH while strictly maintaining the stereochemistry defined in the input SMILES. Final structural refinement was performed *via* energy minimization using the AMBER14 force field [61], optimizing bond lengths and angles to a local energy minimum without altering predefined chiral centers.

The histamine H_2_ receptor (Protein Data Bank (PDB) ID: 7UL3) and the gastric H^+^/K^+^-ATPase (PDB ID: 7W49) were retrieved from the PDB. Target protein structures were prepared by removing all crystallographic water molecules while retaining all other heteroatoms, including structural ions and cofactors, to preserve the native protein fold and structural stability. Polar hydrogen atoms were added, and protonation states were assigned for a physiological pH of 7.4 using YASARA’s automated pK_a_ prediction and experimental neutralization protocols. Final structural refinement was conducted through energy minimization using the AMBER14 force field to resolve steric clashes and optimize the geometry of the target prior to docking. The stereochemical quality of the refined models was validated using the MolProbity web server [62], with backbone dihedral distributions evaluated based on high-accuracy Ramachandran criteria [63]. Corresponding plots and residue statistics are provided in the Supplemental Material (Figures S1 for the histamine H_2_ receptor and S2 for the gastric proton pump).

Docking simulations were conducted using AutoDock [64], as implemented in YASARA Structure. The docking grid was designed to encompass the established binding pockets, guided by the locations of co-crystallized ligands. The grid box was centered at (x, y, z) ═ (--7.619, 8.752, 11.276) Å with dimensions of (15.24 × 17.50 × 22.55) Å for the H_2_ receptor in chain A (7-transmembrane helices), and centered at (x, y, z) ═ (--10.811, 9.935, 9.245) Å with dimensions of (21.62 × 19.87 × 18.49) Å for the potassium-transporting ATPase alpha chain 1 (proton pump). The Lamarckian genetic algorithm was employed with the following parameters: 25 docking runs per ligand, a maximum of 5,000,000 energy evaluations, 27,000 generations per run, and a grid point spacing of 0.375 Å, ensuring the identification of the lowest-energy docked poses. Docking simulations utilized a rigid receptor model with flexible ligand sampling, generating multiple poses for each ligand, which were then clustered by YASARA based on structural similarity to identify representative binding modes. The highest-ranked poses for each ligand were selected for interaction analysis based on predicted binding energy and cluster population. To validate the reliability of the docking setup, we redocked the original co-crystallized ligands, obtaining root-mean-square deviation (RMSD) values of 1.3532 Å for famotidine and 1.5003 Å for soraprazan (Figure S3), indicating that the protocol accurately reproduced the experimental binding modes. Docking results were reported as binding energy (ΔG, kcal/mol), predicted dissociation constants (Kd, µM), and ligand efficiency values (ΔG per heavy atom).

### Calculation of drug-likeness and physicochemical parameters

Drug-likeness and physicochemical parameters were assessed using RDKit [65]. The evaluation included Lipinski’s Rule of Five (molecular weight (MW), logP, hydrogen bond donors (HBD), and acceptors (HBA)), Veber’s rules (topological polar surface area (TPSA) and rotatable bond (RotB) count), and Egan’s filter (logP, TPSA), along with the QED [32]. The QED score integrates descriptors such as lipophilicity, molecular weight, hydrogen bonding capacity, aromaticity, and structural alerts into a single metric, with scores above 0.67 indicative of compounds with generally favorable drug-like profiles. Additional descriptors, including the fraction of sp^3^ carbons, aromatic ring count, and other physicochemical measures, were also evaluated.

### Density functional theory calculations

DFT calculations were performed to investigate the electronic properties of selected coumarin and xanthene derivatives. All calculations were conducted using Spartan 14 software [66]. Geometry optimizations employed the B3LYP functional combined with the 6-31G(d) basis set, without symmetry constraints. The optimized structures were confirmed as true minima on the potential energy surface by the absence of imaginary frequencies.

Frontier molecular orbitals, specifically the Highest Occupied Molecular Orbital (HOMO) and the Lowest Unoccupied Molecular Orbital (LUMO), were calculated for the optimized geometries. The energy gap between the HOMO and LUMO was utilized as a measure of molecular reactivity. Global reactivity descriptors, including chemical potential (µ), global hardness (η), softness (S), and electrophilicity index (ω), were computed from HOMO and LUMO energies as described by Parr et al. [67]. Additionally, dipole moments were calculated for the optimized geometries to characterize the overall molecular polarity of the investigated compounds.

### Prediction of ADMET parameters

The pharmacokinetic and toxicological properties of the marine-derived compounds were evaluated using the ADMETlab 2.0 online platform [68]. This tool predicts key absorption, distribution, metabolism, excretion, and toxicity (ADMET) parameters through machine learning models trained on experimental datasets. For each compound, parameters such as human intestinal absorption (HIA), Caco-2 permeability, plasma protein binding (PPB), blood–brain barrier (BBB) penetration, P-glycoprotein substrate/inhibitor status, major cytochrome P450 (CYP) inhibition potential, and toxicological alerts were computed. The resulting numerical outputs were categorized as favorable, moderate, or risky based on ADMETlab’s internal thresholds, guiding the ranking of compounds for further analysis.

Statistical filtering and comparisons were performed in Python v3.10 using the pandas library, while docking poses, 2D interaction diagrams, and protein–ligand complex visualizations were prepared with Discovery Studio Visualizer v24.1.0.23298.

## Results

**Figure 1.**
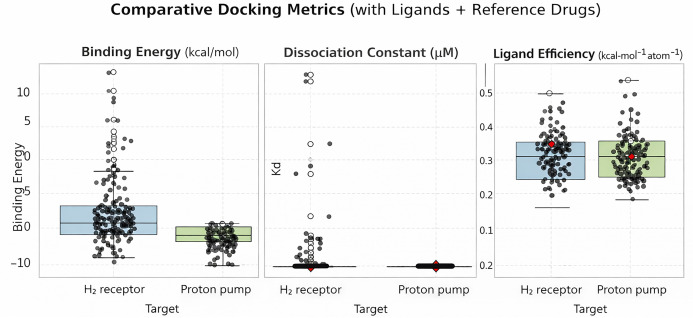
**Comparative distribution of docking-derived metrics for the screened marine library against the histamine H_2_ receptor and the gastric proton pump (H^+^/K^+^-ATPase).** Box-and-whisker plots with overlaid individual data points summarize predicted binding free energies (ΔG, kcal/mol), dissociation constants (Kd, µM; estimated from ΔG), and ligand efficiencies (ΔG per heavy atom, kcal·mol^−^^1^·atom^−^^1^). Each point represents a single ligand from the screening set; boxes indicate the interquartile range with the median, whiskers extend to 1.5×IQR, and open circles denote outliers. Reference drugs are highlighted in red (famotidine for the H_2_ receptor; soraprazan for the proton pump). Abbreviations: H_2_: Histamine H_2_; H^+^/K^+^-ATPase: Gastric H^+^/K^+^ adenosine triphosphatase (proton pump); ΔG: Binding free energy; Kd: Dissociation constant; IQR: Interquartile range.

The molecular docking study of the 161 tested compounds against the H_2_ receptor and gastric H^+^/K^+^-ATPase revealed a wide distribution of binding affinities, dissociation constants (Kd), and ligand efficiencies (Figure 1).

Binding energies (ΔG) obtained from docking serve as a measure of ligand–receptor interaction strength, with more negative values corresponding to stronger and more favorable binding affinities [64]. From these values, estimated dissociation constants (Kd) can be derived, representing the concentration of ligand at which half of the receptor sites are occupied; lower Kd values indicate stronger binding [69]. Beyond binding affinity, predicted ligand efficiency indicates whether a ligand is likely to induce a notable biological effect once bound, distinguishing compounds that bind strongly but may lack activity from those that exhibit meaningful pharmacological potential [70]. Collectively, these parameters facilitate the prioritization of compounds that demonstrate strong binding alongside plausible functional relevance.

Famotidine was selected as the reference ligand for the H_2_ receptor due to its clinical relevance and the availability of a cryogenic electron microscopy (cryo-EM) structure (PDB ID 7UL3) [71]. Soraprazan was chosen as the reference ligand for the gastric H^+^/K^+^-ATPase, as it is a P-CAB that reversibly inhibits the H^+^/K^+^-ATPase at the K^+^-binding site, unlike PPIs that require acid activation and form irreversible covalent bonds [72, 73]. High-resolution structural data for P-CABs, including the soraprazan–pump complex (PDB ID: 7W49), enabled accurate docking and binding-energy comparisons [72]. In contrast, PPIs lack resolved crystal structures with the proton pump due to their transient, pH-dependent activation and covalent binding [73].

When benchmarked against the reference antagonist famotidine (binding energy --7.22 kcal/mol, Kd 5.10 µM, efficiency 0.361 kcal/molAtom), 34 compounds exhibited superior binding energy and Kd (Supplemental Excel File, sheet 1). Docking against the gastric proton pump revealed an even higher proportion of favorable interactions. Relative to the reference drug soraprazan (binding energy --7.95 kcal/mol, Kd 1.49 µM, efficiency 0.294 kcal/molAtom), 98 ligands achieved superior binding energies and dissociation constants (Supplemental Excel File, sheet 2), indicating that members of the library are capable of binding favorably to the proton-pump pocket.

Drug-likeness was assessed using the QED, which integrates several physicochemical descriptors into a single score ranging from 0 to 1. Higher values reflect properties typically seen in approved small-molecule drugs. Compounds with QED values above approximately 0.67 are generally considered to have favorable drug development potential. This metric was utilized alongside traditional filters from Lipinski (MW ≤ 500, logP ≤ 5, HBD ≤ 5, HBA ≤ 10), Veber (TPSA ≤ 140 Å^2^, RotB ≤ 10), and Egan (logP ≤ 5.88, TPSA ≤ 131.6 Å^2^), which evaluate factors such as molecular weight, lipophilicity, hydrogen-bonding capacity, flexibility, and polar surface area. Additionally, the three compounds exhibited zero alerts for Pan-Assay Interference Compounds (PAINS), indicating that their chemical scaffolds do not contain reactive moieties (e.g., quinones, catechols, or ene-rhodanines) typically associated with promiscuous protein binding or redox cycling under assay conditions. Together, these criteria aid in identifying molecules with a balanced physicochemical profile and a reasonable likelihood of favorable pharmacokinetic properties [32].

**Figure 2.**
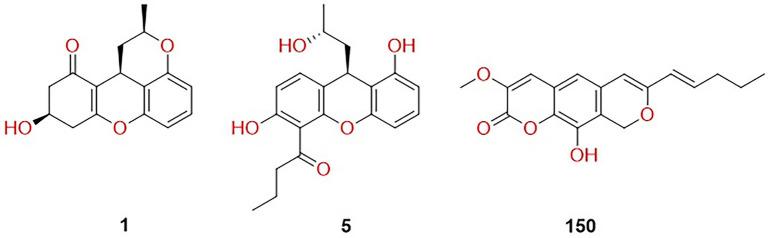
**Chemical structures of the prioritized marine-derived hits (compounds 1, 5, and 150) selected after integrated docking and drug-likeness filtering.** These three candidates outperformed famotidine in the histamine H_2_ receptor docking screen and were the only molecules among the top binders that simultaneously satisfied Lipinski, Veber, and Egan criteria while maintaining QED > 0.67. Compounds 1 and 5 are xanthene derivatives, whereas compound 150 is a pyranocoumarin scaffold; compound 150 also emerged as the most stringent-filtered candidate in the gastric proton pump screen. Abbreviations: H_2_: Histamine H_2_; QED: Quantitative estimate of drug-likeness.

Integration with drug-likeness profiles revealed that only a subset of the previously filtered strong binders complied with multiple medicinal chemistry filters. Specifically, among the 34 marine compounds that surpassed famotidine in binding affinity against the H_2_ receptor, only three met Lipinski’s rule of five, Veber, and Egan criteria while maintaining a QED > 0.67 (Table 1, columns 2, 3, and 4). These compounds represent the most promising candidates for further investigation into H_2_ receptor modulation, combining favorable docking scores with drug-like properties. The high rate of QED > 0.67 in this set suggests a balanced complexity and tractability. The structures of these three compounds are illustrated in Figure 2.

**Table 1:** Docking and drug-likeness parameters of ligands evaluated against the H_2_ receptor and proton pump, exhibiting a QED greater than 0.67

	**1 (H_2_ receptor)**	**5 (H_2_ receptor)**	**150 (H_2_ receptor)**	**150 (proton pump)**
Binding energy [kcal/mol]	--7.49	--7.32	--7.78	--8.57
Dissociation constant [µM]	3.234	4.309	1.982	0.523
Ligand efficiency [kcal/(mol*Atom)]	0.375	0.293	0.338	0.373
MW [g/mol]	272.11	342.15	314.12	
logP	2.31	4.09	3.73	
HBD	1	3	1	
HBA	4	5	5	
TPSA [Å^2^]	55.76	87	68.9	
RotB	0	5	4	
Arom	1	2	2	
Alerts	no	aryl carbonyl type motif	benzopyranone/coumarin-like substructure	
QED	0.787	0.713	0.871	
PAINS	no	no	no	

Abbreviations: MW: Molecular weight; logP: Lipophilicity; HBD: Hydrogen bond donors; HBA: Hydrogen bond acceptors; TPSA: Topological polar surface area; RotB: Rotatable bonds; Arom: Aromatic rings; QED: Quantitative estimate of drug-likeness; PAINS: Pan-Assay Interference Compounds substructures.

Drug-likeness filtering of the 98 marine compounds that outperformed soraprazan in binding affinity against the proton pump narrowed the cohort to only one compound that satisfied Lipinski, Veber, and Egan criteria while maintaining a QED > 0.67 (Table 1, column 5).

Chemical space analysis of the selected ligands indicated that all compounds were located within the classical drug-like region defined by Lipinski, Veber, and Egan criteria, with QED values in the favorable range (≥0.67). Most screened coumarin and xanthene derivatives clustered within a similar range of molecular weight, polarity, and lipophilicity (Supplemental Excel File, sheet 3). These values fall within the range typically observed for many G protein–coupled receptor (GPCR)-active small molecules. When compared to the reference ligands, the marine candidates exhibited greater aromaticity and rigidity than famotidine, but lower bulk and polarity than soraprazan, suggesting that these compounds may achieve reasonable permeability while effectively engaging the receptors.

To enhance the docking results with an electronic-structure perspective, quantum-chemical descriptors were calculated for the three prioritized candidates (compounds **1**, **5**, and **150**) at the B3LYP/6-31G(d) level. The HOMO–LUMO gap (ΔE) and conceptual DFT descriptors (µ, η, S, and ω), alongside the dipole moment, were employed to characterize molecular stability, polarizability, and overall polarity, which can influence electrostatic and hydrogen-bonding contributions to ligand-target interactions (Table 2).

**Table 2:** Calculated quantum chemical parameters for compounds 1, 5, and 150

**Quantum chemical parameters**	**1**	**5**	**150**
Energy (au)	--920.226	--1151.484	--1072.842
E_HOMO_ (eV)	--5.87	--5.62	--5.29
E_LUMO_ (eV)	--1.31	--0.62	--1.74
ΔE (eV)	4.56	5.00	3.55
η (eV)	2.28	2.50	1.78
µ (eV)	--3.59	--3.12	--3.52
ω (eV)	2.83	1.95	3.48
S (eV^--1^)	0.22	0.20	0.28
Dipole moment (D)	2.17	4.14	3.24

**Figure 3.**
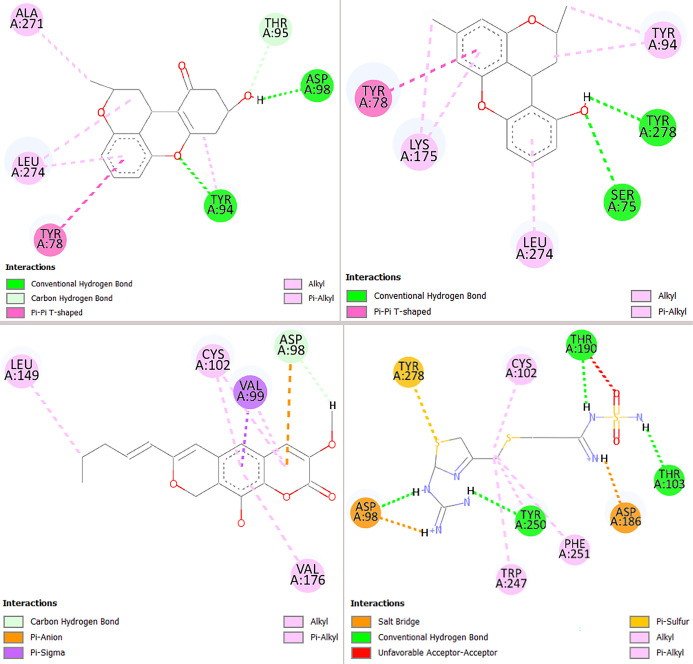
**Protein–ligand interaction maps for prioritized marine hits docked to the histamine H_2_ receptor.** Two-dimensional interaction diagrams are shown for the best-ranked docking poses of compound 1 (top left), compound 5 (top right), compound 150 (bottom left), and the reference antagonist famotidine (bottom right). Compounds 1 and 5 (xanthene derivatives) display closely related binding patterns, sharing aromatic/hydrophobic contacts with Tyr78, Tyr94, and Leu274, but each is stabilized by a single dominant polar interaction (1: Hydrogen bond to Asp98; 5: Hydrogen bond to Tyr278). In contrast, compound 150 (pyranocoumarin scaffold) adopts a distinct pose characterized by π-mediated and hydrophobic stabilization with additional polar anchoring that overlaps with famotidine at Asp98 and Cys102. Interaction types are color-coded as indicated in each panel (e.g., hydrogen bonds, π–π/π-contacts, and hydrophobic contacts). Abbreviations: H_2_: Histamine H_2_; Asp: Aspartate; Cys: Cysteine; Leu: Leucine; Lys: Lysine; Phe: Phenylalanine; Ser: Serine; Thr: Threonine; Trp: Tryptophan; Tyr: Tyrosine; Val: Valine.

**Figure 4.**
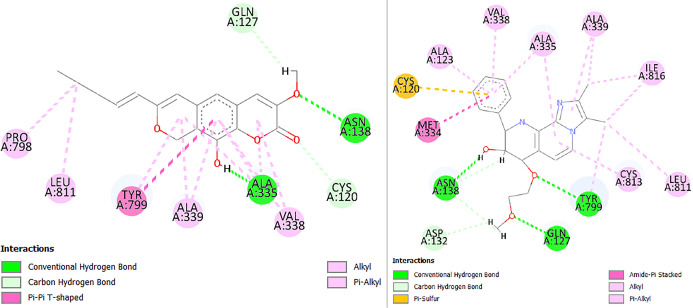
**Comparative interaction maps of compound 150 and soraprazan docked to the gastric proton pump (H^+^/K^+^-ATPase).** Two-dimensional (2D) protein–ligand interaction diagrams are shown for the best-ranked docking poses of compound 150 (left) and the reference potassium-competitive acid blocker soraprazan (right). Compound 150 reproduces a binding pattern closely matching soraprazan within the K^+^-site pocket, sharing a conserved interaction network anchored by Cys120, Gln127, and Asn138, and reinforced by hydrophobic/π-mediated contacts with Ala335, Ala339, Val338, Tyr799, and Leu811. Overall, eight of the nine key residue contacts observed for compound 150 are also present in the soraprazan binding mode, supporting a highly overlapping pose consistent with competitive engagement of the proton-pump pocket. Interaction types are color-coded within each panel (e.g., hydrogen bonds, π-interactions, and hydrophobic contacts). Abbreviations: H^+^/K^+^-ATPase: Gastric H^+^/K^+^ adenosine triphosphatase (proton pump); 2D: Two-dimensional; Ala: Alanine; Asn: Asparagine; Asp: Aspartate; Cys: Cysteine; Gln: Glutamine; Ile: Isoleucine; Leu: Leucine; Met: Methionine; Pro: Proline; Tyr: Tyrosine; Val: Valine.

Figures 3 and 4 illustrate the primary interactions formed by the selected compounds with their respective targets. For the H_2_ receptor, the binding patterns exhibit significant differences compared to famotidine, which aligns with the structural disparities between the molecules. Compounds **1** and **5**, both belonging to the xanthene class, demonstrate similar interaction profiles with the H_2_ receptor (interacting with Tyr94, Tyr78, and Leu274), yet each forms only a single interaction with Asp98 and Tyr278, respectively, similar to famotidine. In contrast, compound **150**, based on a pyranocoumarin scaffold, engages the H_2_ receptor through a distinct set of interactions, overlapping with famotidine at Asp98 and Cys102 (Figure 3).

Additionally, compound **150** exhibits significant overlap in its interaction pattern with soraprazan, screened against the gastric proton pump; eight of the nine key contacts observed for this candidate are also present in soraprazan’s binding mode (Figure 4).

After applying drug-likeness filters and QED thresholds, the remaining compounds underwent absorption, distribution, metabolism, and excretion (ADME) analysis to assess their pharmacokinetic suitability (Table 3). This evaluation focused on properties essential for oral absorption and systemic distribution, including Caco-2 permeability, HIA, plasma protein binding (PPB), and predicted BBB penetration. Interactions with key transporters and metabolic enzymes, such as P-glycoprotein (P-gp) and CYP3A4 or CYP2D6, were also examined. Furthermore, we assessed the likelihood of human ether-à-go-go–related gene (hERG) channel inhibition to estimate potential cardiotoxicity. Collectively, these parameters provided an overview of each compound’s absorption, metabolism, and overall safety profile, aiding in the identification of the most promising candidates. Only a subset of the ADMETlab outputs is reported here; full results are available in the Supplemental Material (Supplemental Excel File, sheet 6). These parameters offered an integrated view of the pharmacokinetic properties and potential safety liabilities of the prioritized compounds, further supporting the selection of candidates with the most favorable absorption and metabolic profiles.

**Table 3:** ADME parameters of optimal candidates selected based on binding affinity and drug-likeness metrics

**Ligand**	**Caco-2 (log cm/s)**	**P-gp I**	**P-gp S**	**HIA**	**BBB**	**PPB**	**CYP** **2C19**	**CYP** **2D6**	**CYP** **3A4**	**hERG**
1	--4.761	0.369	0.979	<0.001	0.292	90.957	0.999	0.001	0.119	0.078
5	--4.840	0.311	0.826	0.009	0.045	95.416	0.630	0.784	0.472	0.098
150	--4.721	0.009	0.002	0.009	<0.001	98.343	0.999	0.959	0.925	0.281
Famotidine	--6.142	<0.001	1.000	0.073	<0.001	10.397	<0.001	<0.001	<0.001	0.281
Soraprazan	--5.352	0.082	0.215	<0.001	0.993	83.806	<0.001	0.002	0.009	0.124

Caco-2 permeability is expressed as log cm/s, with a proper Caco-2 permeability threshold set at > −5.15 log cm/s. The PPB (protein binding percentage) result is represented as a percentage, where a value of **<** 90% indicates that a sufficient amount of the drug remains unbound and is free to reach its target and traverse biological membranes. BBB penetration is also expressed as log cm/s, with logBBB > −1 categorized as BBB+ and logBBB ≤ --1 categorized as BBB--. The output value reflects the probability of a compound being classified as BBB+, ranging from 0 to 1. For the interpretation of HIA, P-gp, CYPs, and hERG results, classification models yield probability values between 0 and 1. Values within the range of 0–0.3 indicate a low probability of negative events (indicating a favorable profile), while values between 0.3 and 0.7 represent moderate or uncertain probabilities. Values from 0.7 to 1 suggest a high probability of negative events, indicating potential issues. Abbreviations: ADME: Absorption, distribution, metabolism, excretion; Caco-2: Caco-2 cell monolayer permeability assay; P-gp: P-glycoprotein; P-gp I: P-glycoprotein inhibition; P-gp S: P-glycoprotein substrate; HIA: Human intestinal absorption; BBB: Blood–brain barrier; PPB: Plasma protein binding; CYP: Cytochrome P450; hERG: Human Ether-à-go-go-Related Gene.

## Discussion

A comparison of results across the two targets reveals a distinct difference in hit distributions. The H_2_ receptor screen yielded fewer high-confidence ligands; however, the top three candidates demonstrated strong compliance with drug-likeness criteria, making them more suitable for medicinal chemistry follow-up. In contrast, the proton pump assay generated a larger pool of raw docking outperformers, yet all but one failed to meet QED or rule compliance. This suggests that while the chemical library contains rich structural motifs capable of engaging the proton pump binding site, optimization for drug-likeness is necessary to advance these scaffolds. Although docking is effective for narrowing down candidates, some high-scoring molecules still lack essential properties for further development. For the H_2_ receptor, a focused shortlist of three compounds can be prioritized for synthesis and biological validation. Conversely, although 98 ligands initially outperformed soraprazan *in silico*, a more stringent filtering process results in only one highly promising candidate for follow-up.

Leptosphaerolide (compound **150**) is a novel, degraded polyketidic lactone isolated from *Leptosphaeria orae-maris* [34]. This fungus is recognized in marine natural product research as a source of secondary metabolites [74]. There is currently no well-documented *in silico*/ADMET prediction for leptosphaerolide in the literature. Due to the limited data on its pharmacology or ADMET profile, leptosphaerolide represents a novel scaffold, less characterized than other candidates, and any *in silico* results should be considered exploratory.

Leptosphaerolide emerged as a dual candidate, demonstrating favorable profiles against both targets. It could serve as a foundation for exploring therapies that simultaneously modulate both pathways. A compound capable of influencing both the H_2_ receptor and the proton pump could be particularly beneficial for patients who continue to experience symptoms despite standard single-target therapies.

Penicixanthene B (compound **5**) was isolated from the mangrove-derived fungus *Penicillium sp*. JY246, obtained from the stem of *Ceriops tagal*. It exhibited insecticidal activity against the larvae of *Helicoverpa armigera* and *Culex quinquefasciatus* [20]. A newer derivative, Penicixanthene E (compound **1**), from the same fungal genus, displayed weak cytotoxic activity against the human pancreatic cancer cell line SW1990 [21]. The pharmacological activity and ADMET characteristics of penicixanthenes in humans have not been thoroughly investigated, indicating that these molecules remain largely uncharacterized and warrant further biological studies.

Since no published studies have investigated marine-derived coumarins or xanthenes as inhibitors of the gastric H^+^/K^+^-ATPase or as histamine H_2_-receptor antagonists, the interpretation of our *in silico* results relies on the closest relevant evidence from terrestrial plant-derived analogues. Reyes-Chilpa et al. reported that several naturally occurring xanthones inhibit the gastric H^+^/K^+^-ATPase, with IC_50_ values ranging from approximately 47 µM to 1.6 mM, indicating moderate but structure-dependent potency [27]. In the same study, the coumarins mammea A/BA and mammea C/OA also exhibited inhibitory activity against the proton pump, albeit with weaker effects (IC_50_ values of roughly 110 µM and 638 µM). The number and position of hydroxyl groups significantly influenced activity, underscoring the sensitivity of the pump to structural variations within these natural-product scaffolds. Although these compounds differ from the marine-derived molecules examined in this study, their reported activity supports the broader relevance of coumarin and xanthene frameworks in modulating gastric acid secretion, providing a useful reference point for interpreting the docking results obtained for the marine metabolites.

The top-scoring compounds in our study shared several physicochemical features: moderate molecular size, balanced polarity and lipophilicity, and a relatively rigid framework. These properties align with those of many GPCR-active small molecules, suggesting their potential to interact with the H_2_ receptor. Structural conservation within GPCR binding pockets, particularly among closely related receptor families, constrains the chemical space available for achieving high selectivity. Ligands optimized for interactions with conserved residues may thus retain affinity across multiple GPCR subtypes, increasing the potential for off-target binding [75].

Moreover, the coumarin- and xanthene-based structures explored here occupy a distinct region of chemical space compared to currently used synthetic antagonists or P-CABs, offering different substitution patterns and oxygen-rich motifs that may interact with the H_2_ receptor and proton pump in ways not accessible to existing drug classes. Collectively, these characteristics position them as promising starting points for further chemical refinement.

A comparison of the docking interaction patterns with the DFT descriptors reveals qualitative rather than quantitative relationships. Compound **150**, which exhibits the smallest HOMO–LUMO gap along with the highest softness and electrophilicity, consistently adopts binding modes driven by complementary hydrophobic and π-mediated contacts, supported by well-positioned polar anchors. In the H_2_ receptor, stabilization involves π-type interactions and electrostatic complementarity with Asp98, while in the gastric proton pump, the ligand engages a compact polar network involving Cys120, Gln127, Asn138, and Ala335, in addition to aromatic stabilization from Tyr799 and hydrophobic packing. This interaction profile suggests greater electronic adaptability and may contribute to the favorable docking scores observed for compound **150** against both targets, including its stronger predicted affinities relative to famotidine and soraprazan.

In contrast, compounds **1** and **5** display wider HOMO–LUMO gaps and lower electrophilicity, consistent with greater electronic stability and more localized interaction networks dominated by hydrogen bonding and steric or aromatic contacts. Although compound **5** shows the highest dipole moment, its binding appears largely shape-driven rather than strongly electrostatically optimized. Overall, these qualitative trends suggest that electronic flexibility may facilitate adaptable binding across different protein environments.

ADME analysis indicated that the selected ligands generally exhibited favorable Caco-2 permeability (> --4.840 log cm/s) and a low likelihood of inhibiting P-gp (0.009–0.369). However, compounds **1** and **5** were predicted to be P-gp substrates (0.979 and 0.826, respectively), which may reduce their intracellular concentrations. Predicted intestinal absorption was high across the set of compounds (0.001–0.009), indicating good oral uptake. All compounds showed low BBB penetration (0.001–0.292), advantageous for non-CNS targets, and exhibited markedly higher plasma protein binding (91%–98%) than famotidine or soraprazan. The predicted CYP inhibition patterns suggest that selected candidates may interact with major metabolic enzymes such as CYP2C19 and CYP3A4. Compounds **1** and **5** displayed lower inhibition probabilities (0.019–0.630) compared to compound **150** (0.925–0.999). Predicted hERG inhibition was low for all molecules (0.078–0.281), indicating a limited risk of cardiotoxicity. Overall, the candidates show encouraging pharmacokinetic features, but their high plasma protein binding and CYP interaction profiles suggest areas requiring optimization to improve exposure and minimize drug-drug interaction risks.

## Conclusion

Our *in silico* results indicate that several marine-derived coumarin and xanthene compounds identified here appear suitable for further investigation as potential modulators of gastric acid secretion. Leptosphaerolide (compound **150**) in particular demonstrated favorable predicted interactions with both the H_2_ receptor and the proton pump, along with acceptable drug-likeness features. Penicixanthenes E and B (compounds **1** and **5**) also exhibited promising properties as H_2_-receptor candidates. The calculated DFT parameters highlight distinct electronic profiles among the compounds and provide qualitative context for interpreting the observed docking trends. In conclusion, the combined use of molecular docking, virtual screening, and drug-likeness/ADMET analyses offers a practical framework for the early identification and prioritization of promising lead compounds, which may be further examined using molecular dynamics simulations. Overall, our findings establish a basis for continued exploration of these scaffolds and may support the development of acid-suppressive compounds with enhanced activity. Future studies should focus on the synthesis of prioritized candidates, *in vitro* binding and enzyme assays, and evaluation in gastric cell lines or *ex vivo* tissue models to confirm their inhibitory potential and biological relevance.

## Supplemental data

Supplemental data are available at the following links: https://www.bjbms.org/ojs/index.php/bjbms/article/view/13660/4115

https://www.bjbms.org/ojs/index.php/bjbms/article/view/13660/4116.

## Data Availability

The datasets generated during and/or analyzed during the current study are available from the corresponding author on reasonable request.
